# Machine learning provides evidence that stroke risk is not linear: The non-linear Framingham stroke risk score

**DOI:** 10.1371/journal.pone.0232414

**Published:** 2020-05-21

**Authors:** Agni Orfanoudaki, Emma Chesley, Christian Cadisch, Barry Stein, Amre Nouh, Mark J. Alberts, Dimitris Bertsimas

**Affiliations:** 1 Operations Research Center, Massachusetts Institute of Technology, Cambridge, MA, United States of America; 2 Office of Innovation, Hartford HealthCare, Hartford, CT, United States of America; 3 Ayer Neuroscience Institute, Hartford HealthCare, Hartford, CT, United States of America; 4 Sloan School of Management, Massachusetts Institute of Technology, Cambridge, MA, United States of America; Universitat Politecnica de Catalunya, SPAIN

## Abstract

Current stroke risk assessment tools presume the impact of risk factors is linear and cumulative. However, both novel risk factors and their interplay influencing stroke incidence are difficult to reveal using traditional additive models. The goal of this study was to improve upon the established Revised Framingham Stroke Risk Score and design an interactive Non-Linear Stroke Risk Score. Leveraging machine learning algorithms, our work aimed at increasing the accuracy of event prediction and uncovering new relationships in an interpretable fashion. A two-phase approach was used to create our stroke risk prediction score. First, clinical examinations of the Framingham offspring cohort were utilized as the training dataset for the predictive model. Optimal Classification Trees were used to develop a tree-based model to predict 10-year risk of stroke. Unlike classical methods, this algorithm adaptively changes the splits on the independent variables, introducing non-linear interactions among them. Second, the model was validated with a multi-ethnicity cohort from the Boston Medical Center. Our stroke risk score suggests a key dichotomy between patients with history of cardiovascular disease and the rest of the population. While it agrees with known findings, it also identified 23 unique stroke risk profiles and highlighted new non-linear relationships; such as the role of T-wave abnormality on electrocardiography and hematocrit levels in a patient’s risk profile. Our results suggested that the non-linear approach significantly improves upon the baseline in the c-statistic (training 87.43% (CI 0.85–0.90) vs. 73.74% (CI 0.70–0.76); validation 75.29% (CI 0.74–0.76) vs 65.93% (CI 0.64–0.67), even in multi-ethnicity populations. The clinical implications of the new risk score include prioritization of risk factor modification and personalized care at the patient level with improved targeting of interventions for stroke prevention.

## Introduction

Over 70% of strokes occur in people without prior history of adverse events, emphasizing the importance of primary prevention [1]. Over the past four decades, several risk scores have been introduced to identify individuals at high risk for cerebrovascular disease [2–4]. These scores highlighted the benefit of introducing blood pressure treatment and other medication, leading to the significant decline of stroke rates over the past 15 years [5, 6].

The Framingham Heart Study Stroke Risk Score (FSRS) is one of the most established and respected standards for estimating 10-year stroke risk [2]. The Framingham Heart Study started with the goal of observing a large population of adults over time to better understand the factors that lead to cardiovascular and cerebrovascular disease. The original FSRS was based on stroke data from the 1960s and 1970s, but its application on contemporary cohorts showed overestimation of stroke risk [7, 8]. Recently, a Revised FSRS (R-FSRS) was introduced to account for temporal trends using data from the offspring cohort and reflecting updated stroke rate incidence [9].

These approaches apply traditional statistical tools such as the Cox Proportional Hazards model [10], which assume a linear, log-linear, or logit-linear relationship between the risk factors and the prevalence of the disease. While useful, they presume that the variables in their models interact in a mere additive fashion. The mathematical and medical realities, however, suggest that the interaction of risk factors and markers of disease acuity are far from linear, and that some variables gain or lose significance due to the absence or presence of other variables [11, 12]. In a logistic regression setting, interactions between risk factors can only be incorporated via cross-multiplication to estimate the combined relative risk. However, this approach requires a significant augmentation of the feature space while it does not generalize to higher numbers of risk factors.

On that ground, we recognized the substantial benefit that algorithmic approaches and machine learning could bring in this field. We propose the Non-linear FSRS (N-SRS) using the clinical examination data from the offspring cohort of the Framingham Heart Study (FHS) to estimate the 10-year stroke risk. To achieve our objective, we utilize novel Machine Learning (ML) methods to predict the progression of cerebrovascular disease [13, 14]. Our model considers a wider spectrum of potential risk factors that include the prescribed medical regimen at the time of the examination. We suggest a new way of utilizing data from longitudinal studies that allows the creation of a larger dataset that can boost the performance of ML methods without introducing bias in the data. Our predictive algorithm is a tree-based method called Optimal Classification Trees (OCT) that allows the physician to explore the exact model and assess the interpretability of its results. Compared with other binary classification methods, such as Neural Networks that are not explainable [15], OCT is comprehensible and can be easily visualized in a tree form [16]. The final model optimally estimates the probability of stroke with superior performance compared to other stroke risk scores. These findings were validated with a separate multiethnic population of 17,527 individuals from an academic medical center.

## Methods

The creation, evaluation, and validation of a new prediction model involves a series of analyses that are necessary to prove its statistical significance. Our methodology comprised the following steps:

1. Identification of the derivation and validation cohort and definition of inclusion criteria. Observations were split into the training (75%) and the testing (25%) sets.
2. Definition of stroke risk factors and outcomes and association with every participant visit included in the data.
3. Imputation of missing values in independent variables using the MedImpute algorithm [14, 25]. Multiple computational experiments were conducted in order to select the most appropriate missing data imputation method.
4. Creation of the Non-linear Framingham Stroke Risk Score using the Optimal Classification Trees algorithm. A risk profile analysis was conducted to validate its insights from the medical literature. The latter was part of an iteration process in tandem with hyperparameter tuning.
5. Training of other ML models using a varied set of supervised learning binary classification algorithms, including Logistic Regression.
6. Discrimination and calibration performance evaluation of all ML models and the R-FSRS for the testing sets of the derivation and validation cohorts. Separate results summary tables and figures were created for each population.
7. Creation of an interactive web-based interface for the communication of the N-SRS model to the clinical community.

### Derivation cohort

Our study sample comprised the Framingham offspring and spouses of offspring cohort enrolled in 1971 and reexamined approximately once every four years since then [17]. To be included, participants were required to be stroke-free and above 40 years of age at each baseline examination. We exclude younger patients following the paradigm of the R-FSRS model [9]. ML methods perform significantly better as the number of the training sample size increases. Thus, we considered for every participant each clinical examination as a distinct observation. We applied the following inclusion criteria:

1. The participant had not experienced a stroke event prior to the date of the baseline clinical examination. Patients with prior history of such adverse events receive specific treatment and their future trajectory highly depends on the severity of their primary stroke. Thus, for these cases we refer the reader to secondary stroke specific risk prediction tools [18].
2. The participant was not censored within 10 years from the time of the clinical examination. For every observation we required that (a) either the participant experienced a stroke within the defined time-frame or (b) the participant was censored after the lapse of 10 years.

This methodology of population sampling resulted in the inclusion of 4,385 unique participants, which translated in 18,793 distinct visits (Table 1 –Framingham Dataset 1 (FD1)). The dataset was split into the training (75%) and testing (25%) population to allow for unbiased evaluation of the algorithms’ performance. Note that visits from the same individual were only included in one of the two sets. Thus, we avoided the introduction of bias in the algorithm evaluation process.

**Table 1:** Baseline characteristics of the derivation and validation populations.

Dataset Name	Parameter	Value
**Framingham Dataset 1 (FD1)**	Sample size	18,793
	Number of participants	4,385
	Number of stroke cases	1,013
	Number of distinct participants with stroke	460
	Proportion of female population	53.97%
**Framingham Dataset 2 (FD2)**	Sample size	2,989
	Number of stroke cases	221
	Proportion of female population	54.26%
**BMC—Caucasian**	Sample size	9,029
	Number of stroke cases	909
	Proportion of female population	58.63%
**BMC–Black**	Sample size	2,862
	Number of stroke cases	230
	Proportion of female population	58.97%
**BMC–Hispanic**	Sample size	5,636
	Number of stroke cases	406
	Proportion of female population	50.19%

### Validation cohort

The stroke risk model was subsequently validated in a prospective multiethnic cohort of 17,527 patients from the Boston Medical Center (BMC), a private, not-for-profit, 487-bed, academic medical center located in Boston, MA, USA. We identified, using the Electronic Health Records (EHR), a stroke-free population at baseline who satisfied the inclusion criteria without censoring (Table 1 –BMC datasets). We retrieved each patient’s medical and family history and formulated a dataset that measured the same characteristics as the Derivation Cohort. Every observation in this population corresponds to a unique patient visit. However, no patient was included more than once in the data set. At least 50% of the independent features were known for all selected samples. Missing values were subsequently imputed using a ML algorithm. Prior visits from the same database were used to identify demographic information or data related to the medical and family history of the patient.

### Definition of stroke risk factors

We used data collated from each clinical examination including all the risk factors considered in the R-FSRS [9], as well as medication, previous treatment information, electrocardiogram (ECG) results, and additional variables considered in other stroke risk scores [17, 19]. Considering the impact of managing blood pressure levels to the progression of cerebrovascular disease, we hypothesized that the inclusion of treatment specific variables could lead to more personalized stroke risk estimation. A full list of all considered independent variables is presented in Table 2. Age, systolic blood pressure (SBP), high-density lipoprotein (HDL) level, body mass index (BMI), hematocrit and fasting blood glucose were treated as continuous features while the rest of the covariates where considered factor variables. SBP was recorded as the mean of 2 physician recorded measurements made on the left arm of the seated subject, using a mercury column sphygmomanometer and a cuff of appropriate width. Baseline CVD was recorded as present if coronary artery disease, congestive heart failure or peripheral vascular disease had been documented in the participant at, or prior to, the clinical examination. Current cigarette smoking was defined as smoking in the year prior to the baseline examination. We used SBP and DBP measurements to define a new variable called “Blood Pressure Category” based on current American Heart Association (AHA) guidelines [20]. We utilized the ECG results provided in each clinical examination of the FHS as additional covariates in our model as well as medical treatment details (i.e. participant underwent CABG or PCI or was under antihypertensive medication at the time et al.). Diabetic status was defined based on the FHS data dictionary similarly to the ECG results. The status of antihypertensive medication was split in 2 levels (0 = no current prescription of antihypertensive treatment, 1 = currently or in the past under antihypertensive treatment). Detailed information about the prevalence of all risk factors at baseline examinations for each cohort can be found at the Supporting Information (S1 Table).

**Table 2:** Stroke risk factors considered in the N-SRS model.

Category	Variable
**Demographic Factors**	Age
	Gender
**Categorical Risk Factors**	Current cigarette smoking
	Presence of Cardiovascular disease
	Presence of Atrial Fibrillation
	History of Transient Ischemic Attacks
	History of Myocardial Infarctions
	Diabetes mellitus
	Blood Pressure Category
**Medication and Treatment related Factors**	Antihypertensive medication
	Statins
	Nitrates
	Diuretics
	CABG
	PCI
**ECG results**	X-ray Enlargement
	Left Ventricular Hypertrophy
	Presence of T-Wave abnormality
	Intraventricular Block
	Atrioventricular Block
	ST-Segment abnormality
	U-Wave abnormality
	Premature beats
**Continuous Risk Factors**	SBP
	HDL
	BMI
	Hematocrit
	Fasting plasma glucose level

### Definition of stroke

Stroke was modeled as a binary outcome and defined as an acute onset focal neurological deficit of vascular etiology, persisting for more than 24 hours, concordant with the World Health Organization (WHO) definition; both ischemic and hemorrhagic strokes were included as in the original FSRS and updated R-FSRS. We used the FHS definition of stroke to specify the outcomes in our dataset; detailed description is defined in previous work [2, 9, 21, 22].

### Ethical oversight

All participants provided informed consent approved by the Institutional Review Board at the Boston University Medical Center for the Framingham Heart Study. The Massachusetts Institute of Technology Institutional and Boston Medical Center Review Boards approved the sharing of data between the two institutions with a research data use agreement. We did not require informed consent by the patients of the Boston Medical Center database as we worked with a HIPAA Limited Data Set.

### Missing data imputation

Missing values were encountered in the majority of the included risk factors. Some participants did not answer the totality of the questionnaires in some of their visits. Moreover, earlier examinations did not record some of the variables, such as echocardiogram results, and thus they were unknown for a subset of the observations [23]. Employing imputation techniques instead of complete case analysis, allows the inclusion of a wider set of features which otherwise would have been omitted by the model [24]. We imputed missing values using a recently developed ML method called MedImpute [14, 25]. The decision to use this algorithm was based on a series of computational experiments that compared both the missing data imputation accuracy as well its effect on downstream predictive performance on these data. It leverages the fact that the same participant could have been included multiple times in the dataset, corresponding to various clinical examinations that satisfied the inclusion criteria. Compared to multiple imputation approaches, such as MICE [26], MedImpute does not require pooling results that affect the interpretability of the final data set. This methodology has been tested to be robust to the particular missing data patterns which are frequently encountered in longitudinal studies [25]. The algorithm outperformed in both imputation accuracy and downstream prediction performance other standard imputation methods, such as mean [27], *k*-Nearest Neighbors [28], OptImpute [29], MICE [26] (Supporting Information, S2 Table). MedImpute reduced the mean absolute imputation error in the Framingham dataset by 5% and increased the c-statistic in the testing set from 85.21% (MICE) to 87.43%. The authors of the algorithm have also done further experiments using data from the Framingham Heart Study under different missing data regimens, including varying levels of missingness from 10% to 50%, increasing number of observations per participant, and different missing data patterns (Missing Completely at Random, Missing Not At Random) [25]. The method was independently applied to the training and testing sets of the Framingham population as well as the BMC cohort.

### Creating the N-SRS

The N-SRS utilizes the Optimal Classification Trees (OCT) algorithm, a novel machine learning method that places emphasis on both accuracy and interpretability [13, 30]. Through this algorithm, we produce a predictive model for 10-year risk of stroke which adaptively changes the splits on the variables, accounting for non-linear interactions among them. The stroke risk is calculated via a series of questions whose order changes dynamically depending on the response. The non-linearity effect is attributed to the absence of a fixed risk coefficient to each independent covariate. The contribution of each feature to the overall score is conditional to other patient characteristics and thus may vary significantly.

Decision tree methods construct a single tree that determines for each observation a single path, or risk profile. This property renders the final output very easy to understand, and thus appropriate for applications where interpretability is important. Its structure allows predictions through a few decision splits on a small number of high-importance variables. This feature is not shared by other ML algorithms such as neural networks or gradient boosted decision trees, which are opaquer and often characterized as “black box” methods [15, 31].

Traditional tree-based algorithms, such as CART [32], take a top-down approach to building a decision tree, applying a greedy heuristic recursively, starting with the full population in the top node and creating each subsequent split in isolation. The CART approach has been criticized because each tree split is determined sequentially without reconsidering the possible impact of future splits in the tree [33]. In practice, this typically leads to decision trees having worse performance than alternative methods [34]. The OCT method was introduced to create the entire decision tree at once through an optimization approach, resulting in more accurate results than its predecessor method [30].

The selection of the final model involved an iterative process during which a risk profile analysis was conducted for each path of the tree. Every path is associated with a unique set of risk factors whose interaction and significance was validated from the medical literature.

We trained other well-established ML algorithms (i.e. CART, Random Forest, XGBoost) on the derivation population data to have a fair comparison of the OCT performance in addition to the R-FSRS results [32, 35, 36]. Logistic regression with L_1_ regularization (Log.Reg) is also employed to specify the performance of a linear model using the same features, data format and missing data imputation as the N-SRS [37]. We used 10-fold cross-validation to set the parameters for each model. The OCT maximum depth was set to eight and the minimum bucket to 20 observations.

### Measurement of model performance

The OCT algorithm performance and its ability to predict 10-year risk of stroke was measured using the c-statistic, also known as the Area Under the Curve (AUC). The AUC measures the ability of a model to discriminate between the outcomes of interest, incorporating both sensitivity and specificity, and has been used as a measure of model success in multiple prior risk-scoring development efforts [38]. We report the average performance across five random partitions of the data with replacement in the derivation population. For each random split, a distinct training sample was used to create the predictive models. Their performance was subsequently evaluated on both the testing sets of the Framingham cohorts as well as the BMC validation cohort. Confidence intervals (95%) were calculated for the bootstrapped results. We also report the average sensitivity, specificity, precision, negative predictive value, positive predictive value for all cohorts and methods when the probability threshold is set to 0.5. In addition, we compare the Hosmer-Lemeshow calibration χ^2^ statistic to measure how closely the outcomes predicted by a given model approximate the observed outcomes [39].

We used three different datasets to measure the performance of the prediction models, including the R-FSRS. In the first set of experiments, we evaluated each model’s outcomes using the testing set of the Framingham Dataset 1 (FD1). The FD1 includes all the clinical examinations of the offspring cohort that satisfied the inclusion criteria but did not participate in the model training process. The Framingham Dataset 2 (FD2) comprises of the observations that the R-FSRS used for its development (Table 1 - FD2). We carefully split the dataset such that observations used in the FD2 are only part of the testing set of FD1. As a result, all reported metrics refer to out-of-sample results. The FD2 does not include any samples from the FD1 training set. We subsequently compared the performance of the N-SRS with the R-FSRS on the validation cohort (Table 1 - BMC) against the same metric.

### Statistical analysis

Our analysis was performed using Julia 1.0 and R version 3.5 [40, 41].

### The user-friendly interface

Leveraging the tree nature of the final N-SRS, we built a dynamic online application as the user-friendly interface of the algorithms for use by clinical providers [42]. The application is in the form of an interactive questionnaire. The questions are adaptive corresponding to risk factors; the subject of each new question depends on the answer to the prior question. When all questions are answered, the user receives the final risk estimate of stroke for the particular patient. The software follows the same interface as the POTTER score, which has been already implemented at the Massachusetts General Hospital, for the estimation of emergency surgery mortality and morbidity risk, with great success [43]. Due to its format, the application could be integrated into an EHR environment, pulling the most available variables directly from the database in an automated fashion. Once integrated into the EHR, the user would only be required to answer questions that cannot be pulled in automatically. If there is full EHR automation, the risk would be calculated at once.

## Results

**Fig 1:**
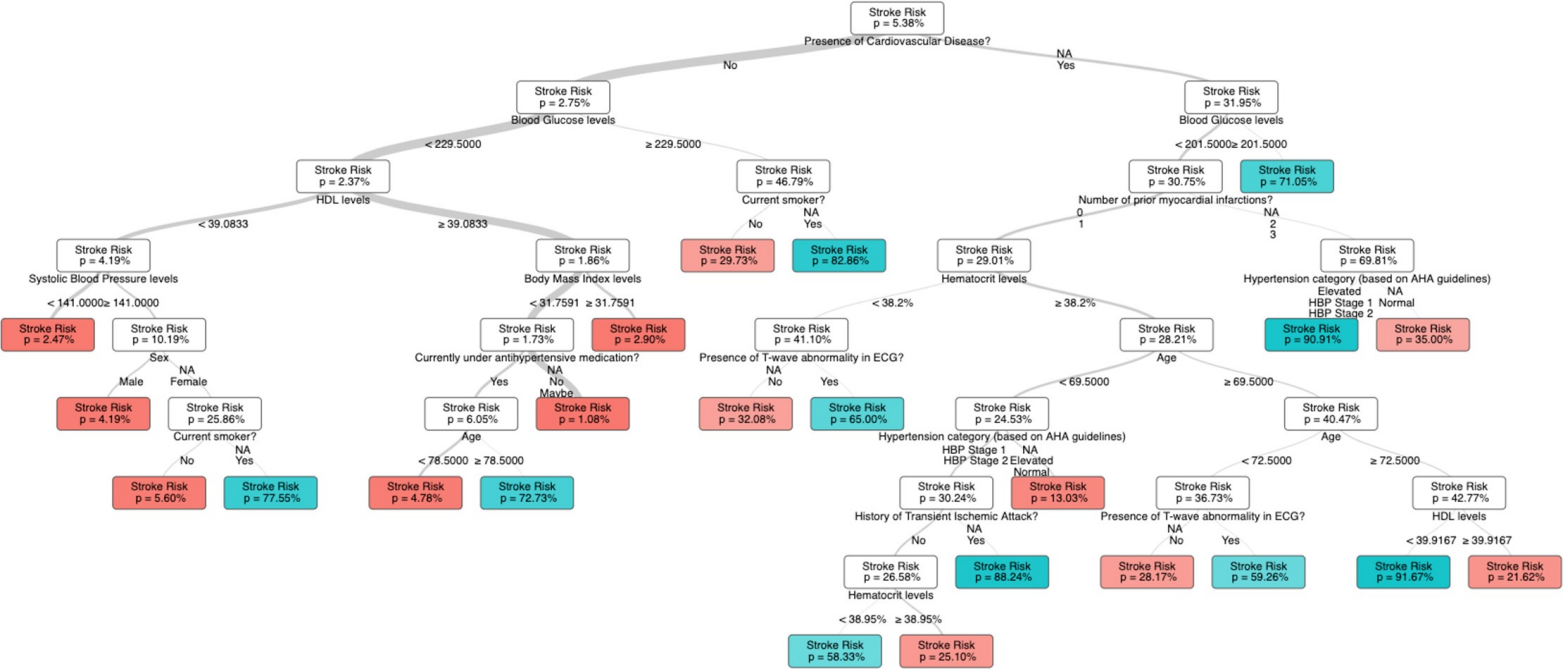
A visualization of the N-SRS tree-based model.

A comprehensive decision-making algorithm was designed, and a user-friendly model, the Non-linear Framingham Stroke Risk Score (N-SRS) was created using the training set of FD1; a total of 14,195 clinical examinations (75%) from the Framingham offspring cohort. Fig 1 provides a visualization of our model in a tree structure. While each node of the tree model reveals important information regarding the associated risk of patients, it should not be considered in isolation. On the contrary, the final risk profile of individuals should be based on the full path until the final “leaf” node of the tree model. Thus, we identify 23 different stroke risk profiles, all of which highlight the effect that these factors might impose in the risk of stroke while introducing new non-linear relationships when combined. Each profile follows a different path of the tree and is affected only by the risk factors that appear in that path (Fig 1).

### N-SRS performance on the Framingham datasets

Table 3 demonstrates the superior performance of the N-SRS compared to the R-FSRS calculator and other established ML methods in both the FD1 and FD2. Notice, that the OCT approach is significantly more accurate compared to the R-FSRS approach leading up to a 15% AUC improvement in DF1 and 9% in DF2 populations, both for male and female. Moreover, the results indicate equivalent performance with respect to other less interpretable ML methods (XGBoost, Random Forest) in the testing set since the absolute difference in the AUC is less than 1%. The Log.Reg models achieve better performance compared to the R-FSRS improving the out-of-sample discrimination metric by 9.37% and 3.55% in FD1 and FD2 respectively. Non-linear ML methods, though, demonstrate superior predictive power that is up to 7.81% (5.06%) higher in the FD1 (FD2) cohorts. The ranking of the methods in terms of downstream performance remains intact between the two datasets. Similar conclusions are also reflected on the sensitivity, specificity, precision, negative and positive predictive value metrics. Receiver Operator Curves (ROCs) are included in the Supporting Information (S1 Fig).

**Table 3:** Comparison of the N-SRS, the R-FSRS, and other machine learning methods performance on the testing set of the Framingham datasets. Reported metrics include sensitivity, specificity, precision, negative predictive value (NPV), and positive predictive value (PPV) at the probability threshold of 0.5. The Table also presents the overall c-statistic (AUC) and calibration χ^2^ results.

	A) Framingham Dataset 1 (FD1)							
	**N-SRS**	**R-FSRS (both genders)**	**R-FSRS (men)**	**R-FSRS (women)**	**Log. Reg**	**CART**	**Random Forest**	**XGBoost**
**Sensitivity**	0.9142	0.8510	0.8461	0.8554	0.8933	0.8802	0.9175	0.9167
**Specificity**	0.7238	0.6902	0.6890	0.7043	0.7102	0.7099	0.7161	0.7354
**Precision**	0.9408	0.9620	0.9353	0.9758	0.9701	0.9736	0.9605	0.9412
**NPV**	0.0592	0.0380	0.0647	0.0242	0.0423	0.0380	0.0863	0.0588
**PPV**	0.9408	0.9620	0.9353	0.9758	0.9621	0.9736	0.9137	0.9412
**AUC**	0.8743	0.7374	0.7188	0.7552	0.8065	0.7981	0.8829	0.8846
**AUC 95% CI**	0.8569–0.9014	0.6976–0.7619	0.6765–0.7636	0.7081–0.8102	0.772–0.8351	0.7676–0.8287	0.8578–0.9081	0.8643–0.9048
**calibration χ2**	1.96	8.05	11.98	5.44	2.88	3.04	1.43	1.58
	B) Framingham Dataset 2 (FD2)							
	**N-SRS**	**R-FSRS (both genders)**	**R-FSRS (men)**	**R-FSRS (women)**	**Log. Reg**	**CART**	**Random Forest**	**XGBoost**
**Sensitivity**	0.8948	0.8533	0.8605	0.8487	0.8763	0.8504	0.8938	0.8934
**Specificity**	0.5097	0.4217	0.4066	0.4800	0.4867	0.2505	0.4994	0.5110
**Precision**	0.9693	0.9617	0.9531	0.9712	0.9688	0.9393	0.9816	0.9700
**NPV**	0.3973	0.2233	0.2321	0.1933	0.2576	0.1704	0.3804	0.4053
**PPV**	0.9693	0.9617	0.9531	0.9712	0.9401	0.9486	0.9535	0.9700
**AUC**	0.8238	0.7488	0.7281	0.7677	0.7754	0.6884	0.8216	0.8260
**AUC (95% CI)**	0.791–0.8558	0.7145–0.7831	0.6775–0.7788	0.7149–0.8204	0.738–0.8119	0.6435–0.7333	0.7881–0.8536	0.7938–0.8567
**calibration χ2**	2.75	7.3	12.1	4.1	6.5	20.34	2.81	2.7

**Fig 2:**
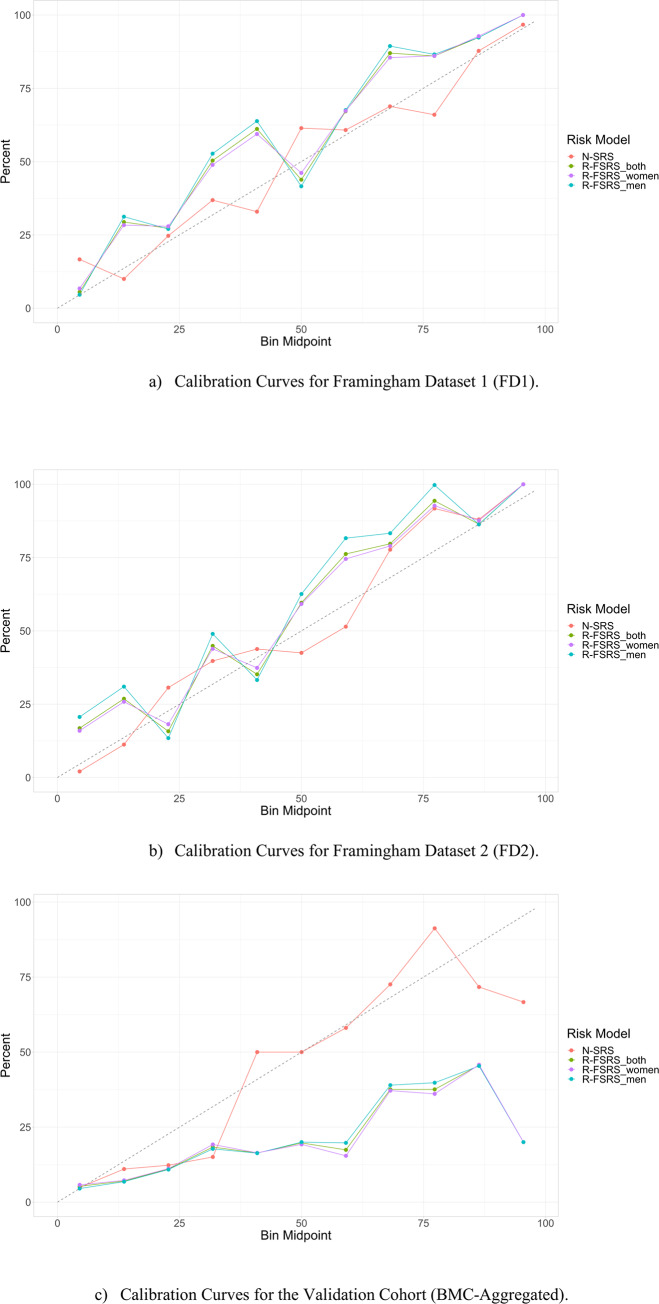
Calibration plots for all models on the Derivation cohort. Fig 2A refers to the testing population of FD1 and Fig 2B to FD2. The plots show the relation between the true class of the samples and the predicted probabilities. Samples were binned to their class probabilities generated by the model. The following intervals were defined: [0,10%], (10,20%], (20,30%], … (90,100%]. The event rate for each bin was subsequently identified. For example, if 4 out of 5 samples falling into the last bin are actual events, then the event rate for that bin would be 80%. The calibration plot displays the bin mid-points on the x-axis and the event rate on the y-axis. Ideally, the event rate should be reflected as a 45° line.

Most importantly, the N-SRS is able to better estimate the true risk of stroke, at different levels of risk. Its Hosmer-Lemeshow calibration χ^2^ statistic is 1.96/2.75 (FD1/FD2) for 8.05/7.3 the N-SRS and R-FSRS respectively. We constructed calibration curves for our models, where best performance is represented by a slope of 45°. The R-FSRS models suffered a decline in calibration, especially at medium risk predicted probabilities. The N-SRS classifier appeared to have the best calibration across all levels. The calibration curves are depicted in Fig 2 for the N-SRS and R-FSRS. The reader can find the corresponding graphs for Log.Reg, CART, Random Forest, and XGBoost in the Supporting Information (S2 Fig).

### N-SRS performance on the validation cohort

Table 4 shows an overview of the results for the N-SRS, R-FSRS, and other ML methods on the Validation Cohort. The non-linear approach (N-SRS) improves the aggregated stroke risk AUC by 16.17% for men and 10.59% for women upon the R-FSRS. Similar results are also recorded in the ethnicity-specific populations. We notice that both stroke risk scores are less accurate in the BMC dataset compared to FD1 and FD2 (-7.09% N-SRS, -8.00% R-FSRS). However, the N-SRS is more robust to other sources of data. Its performance is less affected compared to the R-FSRS. The performance of other ensemble ML algorithms is equivalent to the N-SRS providing an edge of 0.8–0.91%. The Log.Reg models improve upon the R-FSRS by 5.55% but is still weaker than the N-SRS by 3.38%. Table 5 shows that the predictive accuracy of our model remains the same between the Caucasian and the Black population (~74.5%) and gets slightly negatively impacted in the Hispanic population (72.8%). All other ML models achieve higher performance in the Caucasian sample compared to other ethnicity sub-populations.

**Table 4:** Comparison of the N-SRS, the R-FSRS, and other machine learning methods performance on the Validation cohort. Reported metrics include sensitivity, specificity, precision, negative predictive value (NPV), and positive predictive value (PPV) at the probability threshold of 0.5. The overall c-statistic (AUC) and calibration χ^2^ results are also presented. The results refer to the aggregated population.

	N-SRS	R-FSRS (both genders)	R-FSRS (men)	R-FSRS (women)	Log. Reg	CART	Random Forest	XGBoost
**Sensitivity**	0.8986	0.8403	0.8411	0.8396	0.8576	0.8402	0.9055	0.9076
**Specificity**	0.4019	0.3663	0.3786	0.3565	0.3733	0.3599	0.4078	0.4092
**Precision**	0.9395	0.9320	0.9329	0.9313	0.9349	0.9348	0.9407	0.9455
**NPV**	0.2771	0.1815	0.1882	0.1762	0.2026	0.1805	0.2811	0.2818
**PPV**	0.9395	0.9320	0.9329	0.9313	0.9345	0.9317	0.9421	0.9446
**AUC**	0.7403	0.6491	0.6246	0.6735	0.7065	0.6829	0.7482	0.7501
**AUC (95% CI)**	0.7149–0.771	0.6266–0.6716	0.5931–0.6555	0.6411–0.7058	0.6772–0.7558	0.6484–0.7175	0.7198–0.7801	0.7202–0.7856
**calibration χ2**	7.12	36.66	37.42	35.98	25.03	35.76	6.67	6.52

**Table 5:** Comparison of the N-SRS, and the R-FSRS performance on the Validation population using the c-statistic. Detailed results are shown for the main ethnicity groups.

	BMC–White		BMC–Black		BMC–Hispanic	
Model	AUC	95% CI	AUC	95% CI	AUC	95% CI
**N-SRS**	74.30%	0.7149–0.771	75.80%	0.7345–0.767	72.79%	0.6889–0.7671
**R-FSRS (both genders)**	64.91%	0.6266–0.6716	64.85%	0.6304–0.6666	61.04%	0.5601–0.6587
**R-FSRS (women)**	67.35%	0.6411–0.7058	65.22%	0.628–0.6764	61.06%	0.5548–0.6663
**R-FSRS (men)**	62.46%	0.5931–0.6555	64.49%	0.6181–0.6717	61.01%	0.5621–0.6587
**Log.Reg**	71.55%	0.6823–0.7402	69.77%	0.6823–0.7402	70.46%	0.6765–0.7359
**CART**	69.01%	0.6627–0.7134	66.41%	0.6272–0.6609	66.10%	0.6286–0.6934
**Random Forest**	75.08%	0.7162–0.7855	73.14%	0.7139–0.749	70.80%	0.6807–0.7354
**XGBoost**	77.32%	0.7582–0.7881	74.88%	0.7133–0.7842	74.27%	0.7187–0.7667

The calibration statistic demonstrates an edge of N-SRS (7.12) over the R-FSRS for both women (35.98) and men (37.42), following a similar trend to what was shown for the Framingham datasets. Fig 2 shows that the R-FSRS is associated with poor identification of true risk for groups higher than 30%. The corresponding graphs for Log.Reg, CART, Random Forest, and XGBoost are available in the Supplementary material (S2 Fig). In terms of sensitivity and sensitivity, we found that the N-SRS model achieved up to 89% and 40%, respectively while R-FSRS achieved 84% and 36.6%.

## Discussion

To the best of our knowledge, this is the first validated non-linear, interpretable stroke risk predictor that outperforms the established R-FSRS, providing additional insightful information. Overall, our results demonstrate the superior capability that sophisticated ML methods and data utilization can bring in adverse event prediction when coupled with data from large population cohorts. In our ever-changing medical landscape, linear models that entail an additive effect for each known risk factor do not answer many practical questions faced by patients. Patients with multiple medical comorbidities may not be reflected with traditional risk stratification scores such as the FSRS. The NSRS methodology has introduced novel risk factors that are associated with stroke incidence. Moreover, a “one size fits all” approach may not work for a particular patient. Although correlative, the superior interpretability of the model can allow for better patient education when addressing risk factor modification strategies.

Khosla et al and colleagues have previously demonstrated the superiority of ML over cox-hazard methods for stroke prediction with an AUC as high as 0.777 utilization patient data from 5201 patients from the cardiovascular heart study between 1989–1999. Several novel risk factors were identified using this methodology including total medications, maximal inflation level, general health and any ECG abnormality [44]. In contrast to this paper, our methodology utilized interpretable OCT and utilized a robust data set (the Framingham heart study) therefore risk factors where more specific (T-wave abnormality on EKG as compared to “any ECG abnormality) making its utility more relevant.

Other novel ML methods have evaluating stroke risk in specific high-risk populations. Letham et al., developed and interpretable and accurate model for stroke risk prediction in patients with atrial fibrillation utilizing the Bayesian Rule List (BRL) model in contrast to the established linear prediction scores; the CHADS2 and CHA2DS2-VASc risk scores [45]. In this study, claims data from the MarketScan Medicaid Multi-State Database was utilized to study a patient with diagnosis of atrial fibrillation (one year of observation time prior to the diagnosis and one year of observation time following the diagnosis) yielding 12,586 patient with 1786 (14%) suffering a stroke within a year of the atrial fibrillation diagnosis. The BRL performance had a higher performance by AUC as compared to the CHADS2, CHA2DS2-VASc and CART methods (0.756 vs. 0.721, 0.677 and 0.704) respectively. However, as known with claims data and coding, the true interpretability of this methodology is questionable. For example, the BRL states: “if cerebrovascular disorder then stroke risk 47.8% (44.8%–50.7%) else if transient ischemic attack then stroke risk 23.8% (19.5%–28.4%) else if occlusion and stenosis of carotid artery without infarction then strokerisk15.8% (12.2%–19.6%)”. These terms are non-specific and descriptive at best and do not mean anything from a physician perspective. The terms transient ischemic attack and occlusion and stenosis of carotid artery without infarction are both similar clinically, and interchangeable from a coding perspective and cannot be used to risk stratify adequately.

Primary prevention targeting stroke risk factors have been effective in reducing stroke morbidity and mortality in generalized populations [46]. However, they do not consider the potential to predict which of the risk factors would affect each individual and lead to stroke occurrence; a key element in practical disease prevention, targeted therapy and the most compelling finding of our study. Our approach introduces tree-based decision rules where the number of variables required to determine the stroke risk profile is not fixed by our preconceived understanding of comorbidities and attributable risk [46].

The N-SRS model was developed using the Framingham data, a well-established longitudinal data set in contrast to static datasets typically utilized for risk prediction [47]. The model established several key branching points in the tree that confirm the medical validity of this model as well as novel points uncovering new medical insights that had not been evaluated for stroke risk in the past. It also demonstrates the correlation of interplay between risk factors and weighted relevance they may possess in contrast to the binary effect they carry.

The model was validated using an external independent cohort comprised of diverse ethnicities. Our results revealed a superior performance of the N-SRS over the R-FSRS in the training and validation population for both women and men. Additional experiments show that other less transparent non-linear algorithms achieve equivalent performance. Logistic regression models using the same data pre-processing and training sample improve upon the N-SRS but do not outperform more sophisticated ML methods. We hypothesize that the performance of the latter is improved compared to the R-FSRS due to the higher sample size, larger number of features, and the application of an advanced missing data imputation algorithm. Since the accuracy of the N-SRS was higher and more robust to populations from other ethnicities, our model can be generalized with higher degree of confidence compared to the existing stroke risk score. We believe that the increased accuracy of N-SRS is due to the introduction of a larger sample size, new risk factors, and new missing data imputation and binary classification methodologies.

Our proposed way of leveraging the longitudinal study data avoids the induction of bias in the model due to its clear delineation between the training and the testing population. We strictly require that observations from the same individual belong in at most one of these two sets, avoiding potential natural boosts in the downstream performance. Moreover, our results from the multi-ethnicity validation cohort of the BMC demonstrate that the N-SRS generalizes better than its predecessor (R-FSRS).

The main benefit of using decision trees over other methods is their interpretability which, in applications such as healthcare. This attribute is not only essential but often preferred over the maybe higher accuracy that other, non-interpretable, methods may offer [48]. In our models, we show that less transparent, “black-box” algorithms have comparable performance to our suggested model. The latter offers the physician the opportunity to evaluate the risk profile itself and assess the correlation of risk factors relevant for each patient. It also addresses concerns related to the transparency and fairness of the model [49].

Known findings that appeared as branching nodes in the N-SRS include patients with the lowest stroke risk profile being non-diabetic with HDL levels > 39.1 mg/dl and non-hypertensive with an approximately 1% 10-year stroke risk. In contrast, patients with history of cardiovascular disease, diabetes and hypertension carry a 90% stroke risk over 10 years (Fig 1). Of note, these modifiable risk factors weigh heaviest and are independent of other concomitant factors or non-modifiable ones such as age or gender. In fact, the relevance of gender was only pertinent in a subset of patients with no cardiovascular disease or diabetes but with hypertension and low HDL levels.

**Fig 3:**
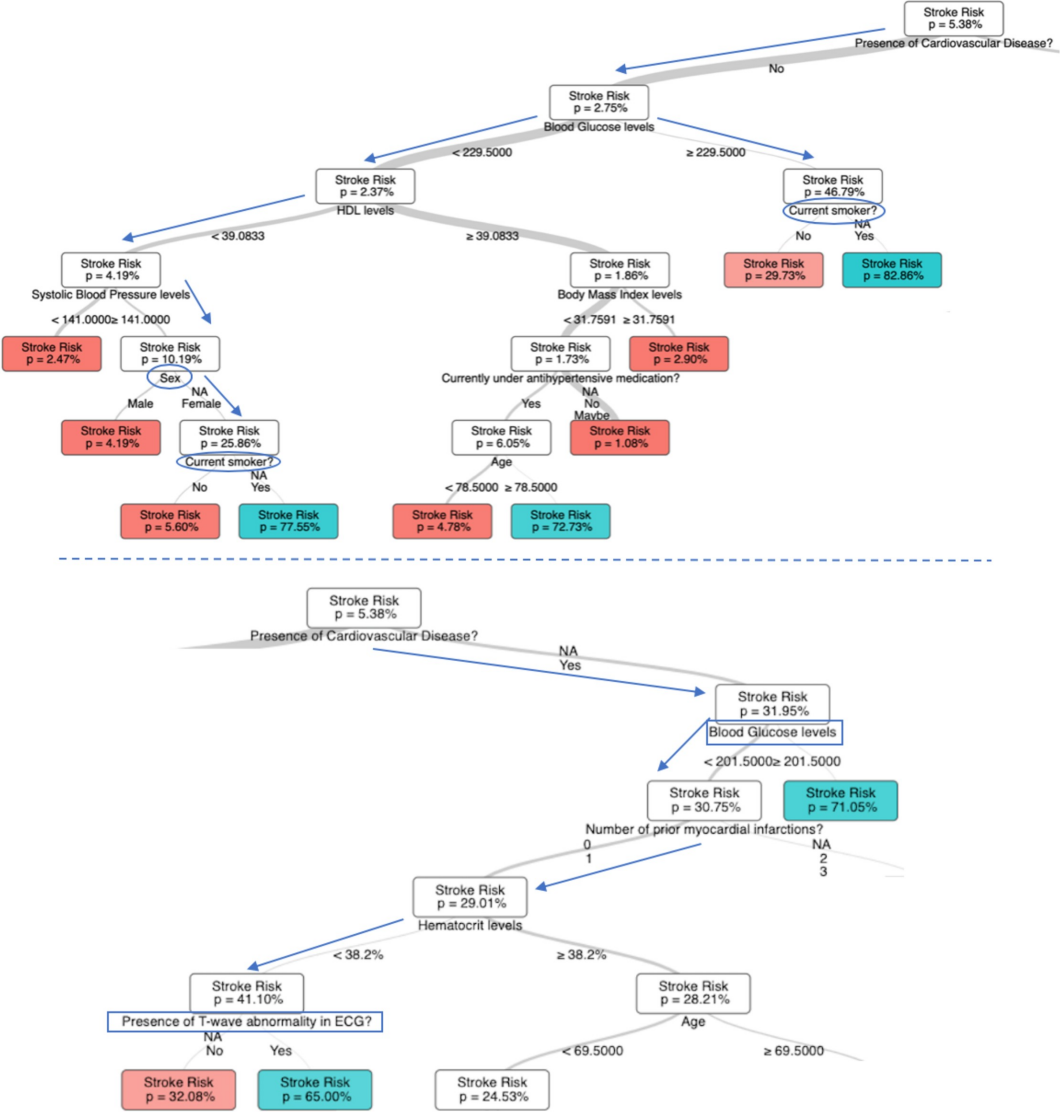
Deep-dives in insightful risk profiles of the N-SRS model.

Note that in some cases to characterize the risk of stroke for certain profiles of the population only three to two variables might be relevant. For people with no history of cardiovascular disease and diabetes, smoking affects dramatically their risk projection increasing the overall stroke score from 29.73% to 82.66% (Fig 3). We notice also that for patients with prior history of cardiovascular disease diabetes is the defining factor of their stroke risk increasing it to 71.05% from 31.95% (Fig 3). The presence or absence of any other risk factor does not influence the overall prediction of the ML algorithm.

An illustration of novel findings includes the relevance of T-wave abnormality on ECG and hematocrit levels in a patient’s 10-year stroke risk profile. For example, the association of major and minor ST-T wave abnormalities on ECG and associated stroke risk has been previously evaluated in a small cohort of Japanese patients but found to be only relevant in men with minor ST changes and both genders for major ST changes based on the small sample size. Furthermore, stroke risk was reduced after adjusting for hypertension [50]. Therefore, the applicability is minimal in evaluating preventative strategies and guiding patient education or intervention. In the N-SRS model, T wave abnormalities were pertinent in some scenarios. A characteristic case refers to patients with history of cardiovascular disease, non-diabetic, with 0–1 MI events, and HCT levels of <38.2% where the 10-year stroke risk changes from 32% to 65% in the absence or presence of T-wave abnormalities respectively (Fig 3).

Such assessments of risk factors and their respective weighted relevance could not be established by linear methodologies and can explain innumerable circumstances where patients may have or lack traditional risk factors and either develop strokes or not. This is the key to personalizing a customized approach to primary prevention.

For instance, the N-SRS shows that the 10-year stroke risk is actually dramatically impacted by smoking changing from 5% to 77.5%. If this patient was not hypertensive in the first place, her 10-year stroke risk would be 2.5% and smoking would not drive this number (Fig 3). This validated risk prediction can highly impact the patient and provider understanding of stroke risk factor associated with incidence for effective guided counseling given the precious resources and time available to practitioners and patients.

Although this is the first validated interpretable machine learning model applied to stroke for 10-year risk prediction, similar applications in other disease entities provided insights obscured by traditional linear methodology and therefore influence personalized care. Bertsimas and colleagues recently evaluated outcomes of 13 different medication regimen therapies in over 10,000 patients with type 2 diabetes and predicted change in target glycated hemoglobin A1c levels [51]. In this model, patients where a suggested change in therapy based on the machine algorithm was made, a predicted reduction by close to 0.5% points in Ac1c was observed. Similar mortality and morbidity risk calculators have also been introduced in the areas of elective surgery, oncology, and transplantation with great success [34, 43, 52]. Such ML-based algorithms can drive personalized medicine and influence outcomes.

**Fig 4:**
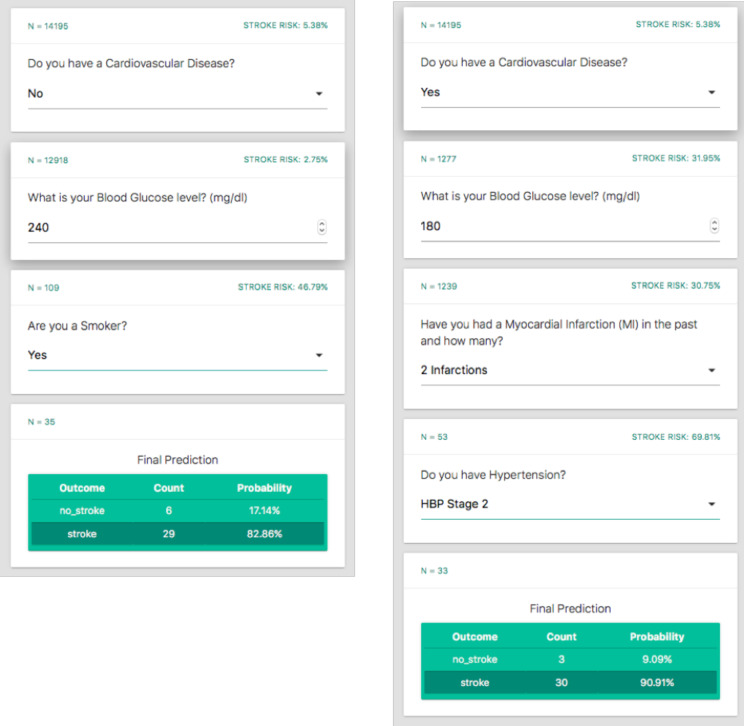
An example illustrating the user-friendly interface of N-SRS. Due to its interactive nature the answer to a question dictates the next question. In this specific example, whether the provider answer yes to no to the question regarding CVD takes the algorithm and questions in a different direction.

We have created an interactive web-based interface through a series of short specific yes and no questions (link) to improve efficiency and usability of the N-SRS decision-tree (Fig 4) [42]. A user’s answer to the first question will dictate what the next grouping the results into 23 categories of risk profiles. Each interaction with the application corresponds to a unique decision-tree node and is based on the specific patient characteristics.

As a second phase of this study, we intend to prospectively follow a patient population in the primary care setting utilizing the N-SRS to guide preventative strategy. In this prospective study, we will not only be able to study real-time prospective stroke risk, but also a completely novel experience of personalized stroke risk assessment care and intervention. This has not been effectively studied in patients at risk for cerebrovascular disease and opens many potential possibilities for other cerebrovascular diseases other than stroke.

### Limitations

The key limitation of our model is the use of input data solely from the FHS which is a Caucausian population. Moreover, there is potentially lack of generalizability to populations from other geographic regions in the United States as well as internationally, and socioeconomically different populations from those of the FHS or BMC. Even though we validate our results in a multi-ethnicity population, we believe that we will need to retrain our algorithm with data from other longitudinal studies and not only EHR.

The validation population is based solely on hospital records and as a result it tends to be sicker than the Framingham cohort. Each observation corresponds to a unique patient visit. Thus, the presence of a patient in the data set is mostly correlated with how detailed was the clinical examination during the visit and if there was any family or personal history recorded in the past at the same clinic.

In addition, we would like to stress that our data is not independent and identically distributed. However, we believe that no bias has been introduced in the training process since both the accuracy and the calibration of the N-SRS is significantly higher than the R-FSRS in both the Framingham 2 dataset and the validation cohort from BMC. Another limitation refers to causality between the variables and the outcomes, which is still not proven despite the high degree of association connectivity between the two. The performance of N-SRS has not been directly compared to other stroke risk functions, such as the CHADS_2_ or the CHA_2_DS_2_-VAS_c_ score for atrial fibrillation stroke risk [53]. Future work could leverage other validation populations to relate the N-SRS predictive performance with these studies.

We also acknowledge prospective validation of this model would outperform validation of blinded data sets, and provide insights beyond performance such as adoption among healthcare providers, interpretability for patients and effects on primary prevention strategies and counseling. A prospective trial design is currently under evaluation.

### Conclusions

We have developed N-SRS, an accurate stroke risk calculator that outperforms, in accuracy and user-friendliness, the existing stroke risk prediction tool. N-SRS might prove useful as an evidence-based, adaptive, and interactive risk calculator tool for primary prevention of stroke. Further studies are needed to explore the ability of N-SRS to predict the occurrence of stroke in other populations. Future work will focus on defining the N-SRS risk levels that warrant therapeutic treatment for primary stroke prevention similar to that available for the primary atherosclerotic cardiovascular disease prevention.

## Supporting information

S1 Fig Receiver Operator Curves (ROC) assessing the discrimination for incident stroke of the N-SRS, R-FSRS for women, R-FSRS for men, CART, Random Forest, XGBoost for the Framingham datasets. (DOCX)

S2 Fig Calibration curves of the N-SRS, R-FSRS for both, R-FSRS for women, R-FSRS for men, CART, Random Forest, and XGBoost for the Framingham datasets. (DOCX)

S1 Table Prevalence of all risk factors at baseline examination for each cohort. (DOCX)

S2 Table Imputation algorithm comparison for the Framingham 1 dataset. The imputation accuracy was measured when the missingness percentage is fixed at 40%. Artificial values were introduced under the Missing Completely at Random pattern to measure the Mean Absolute Error (MAE) and the Root Mean Squared Error (RMSE). Predictive accuracy was assessed for an OCT model trained on the imputed data on the 10-year risk of stroke task. All metrics reflect the average value across five bootstrapped splits of DF1 in training and testing set.
(DOCX)

S3 Table Stroke risk factors identified in the N-SRS algorithm. (DOCX)
