# Supplementary material for: Machine learning provides evidence that stroke risk is not linear: The non-linear Framingham stroke risk score
Source: PLoS One. 2020 May 21;15(5):e0232414. doi: 10.1371/journal.pone.0232414 (PMC7241753; doi:10.1371/journal.pone.0232414)
Supplement: S1 Fig — (DOCX) [file pone.0232414.s001.docx]

**S1 Figure: Receiver operator curves (ROC) assessing the discrimination for incident stroke of the N-SRS, R-FSRS for women, R-FSRS for men, CART, Random Forest, XGBoost for the Framingham Datasets.**


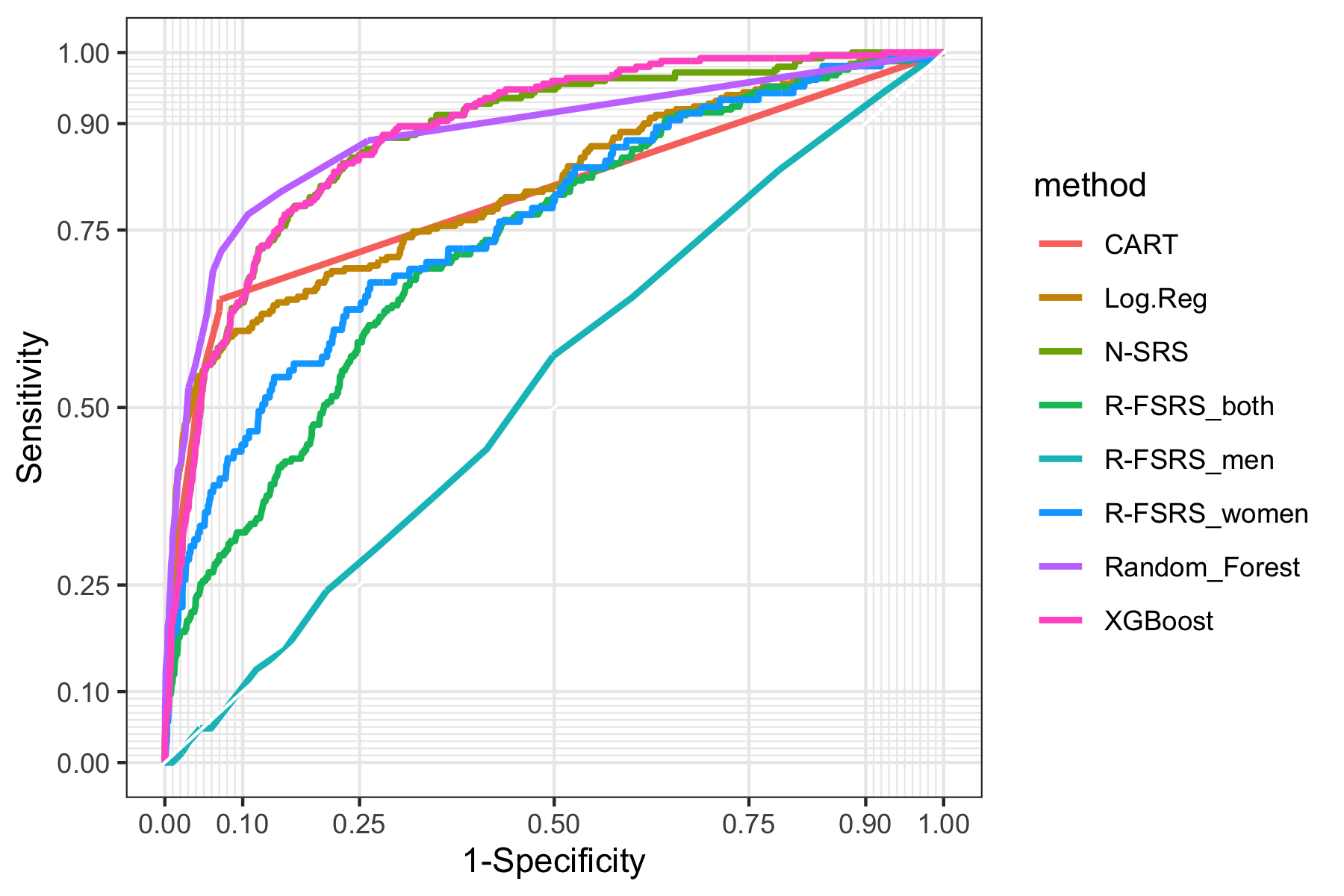


1. ROC curves for Framingham Dataset 1 (FD1).


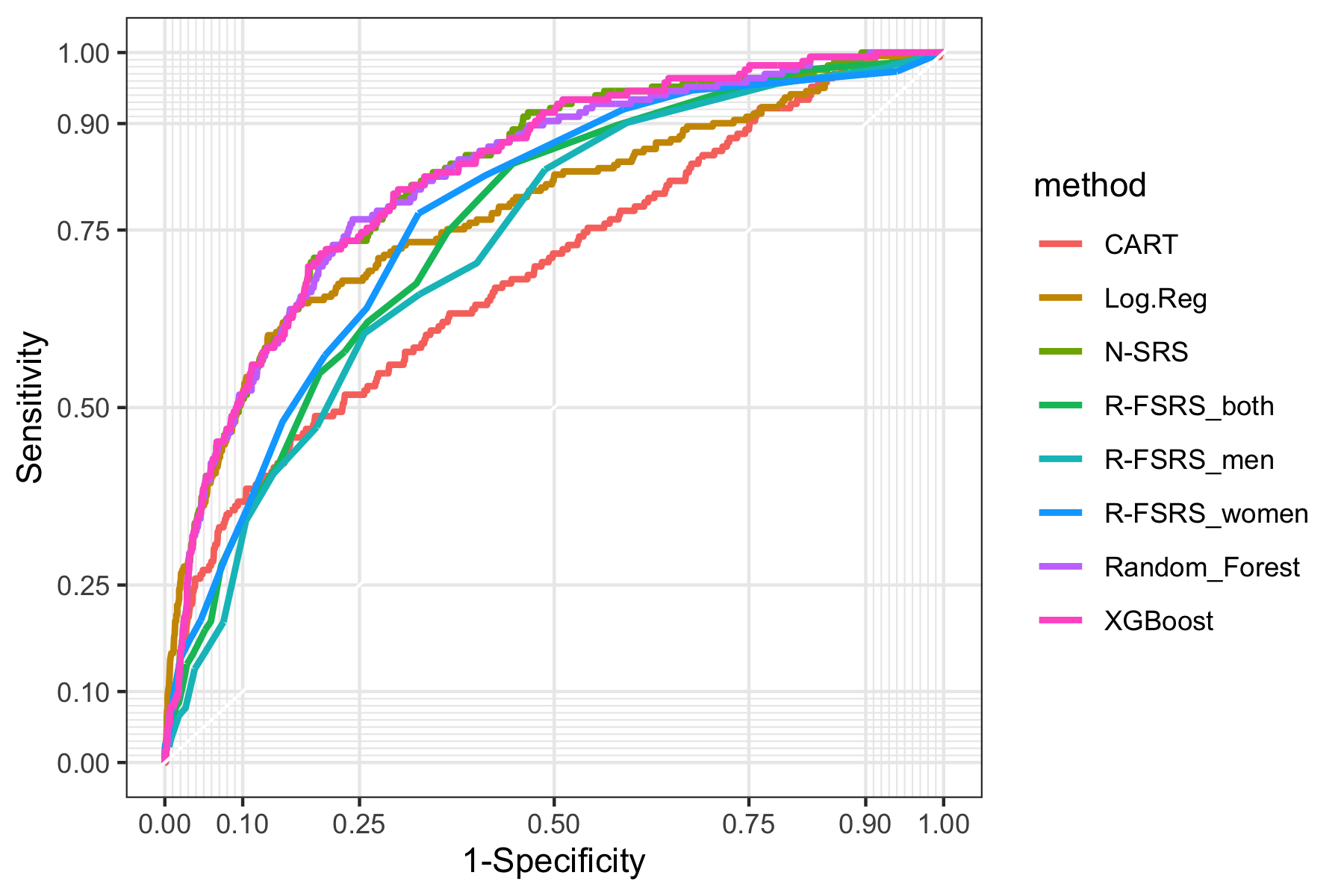


1. ROC curves for Framingham Dataset 2 (FD2).


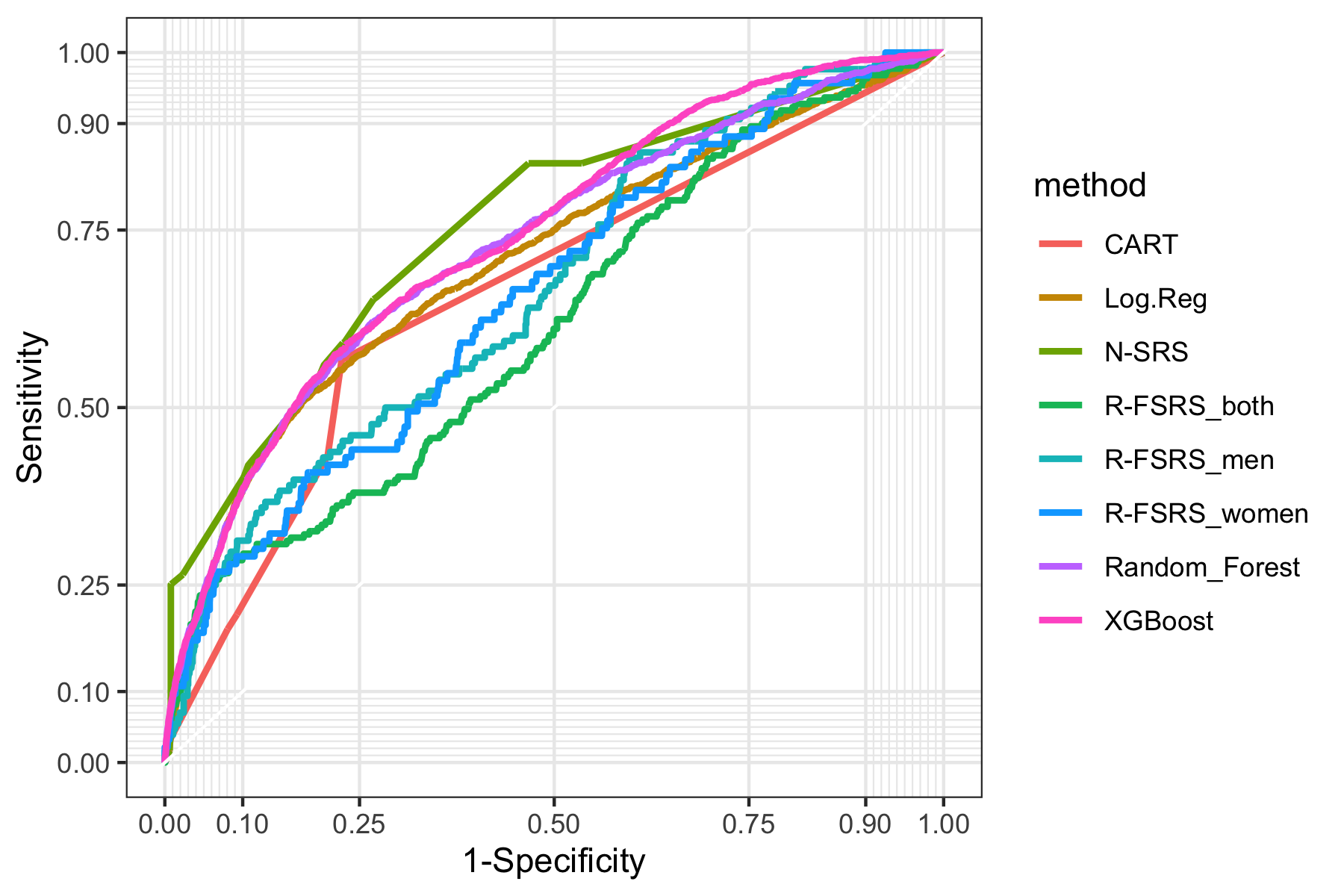


1. ROC curves for the BMC Validation Cohort.
