# Supplementary material for: Machine learning provides evidence that stroke risk is not linear: The non-linear Framingham stroke risk score
Source: PLoS One. 2020 May 21;15(5):e0232414. doi: 10.1371/journal.pone.0232414 (PMC7241753; doi:10.1371/journal.pone.0232414)
Supplement: S2 Fig — (DOCX) [file pone.0232414.s002.docx]

**S2 Figure: Calibration curves of the N-SRS, R-FSRS for both, R-FSRS for women, R-FSRS for men, CART, Random Forest, and XGBoost for the Framingham Datasets.**


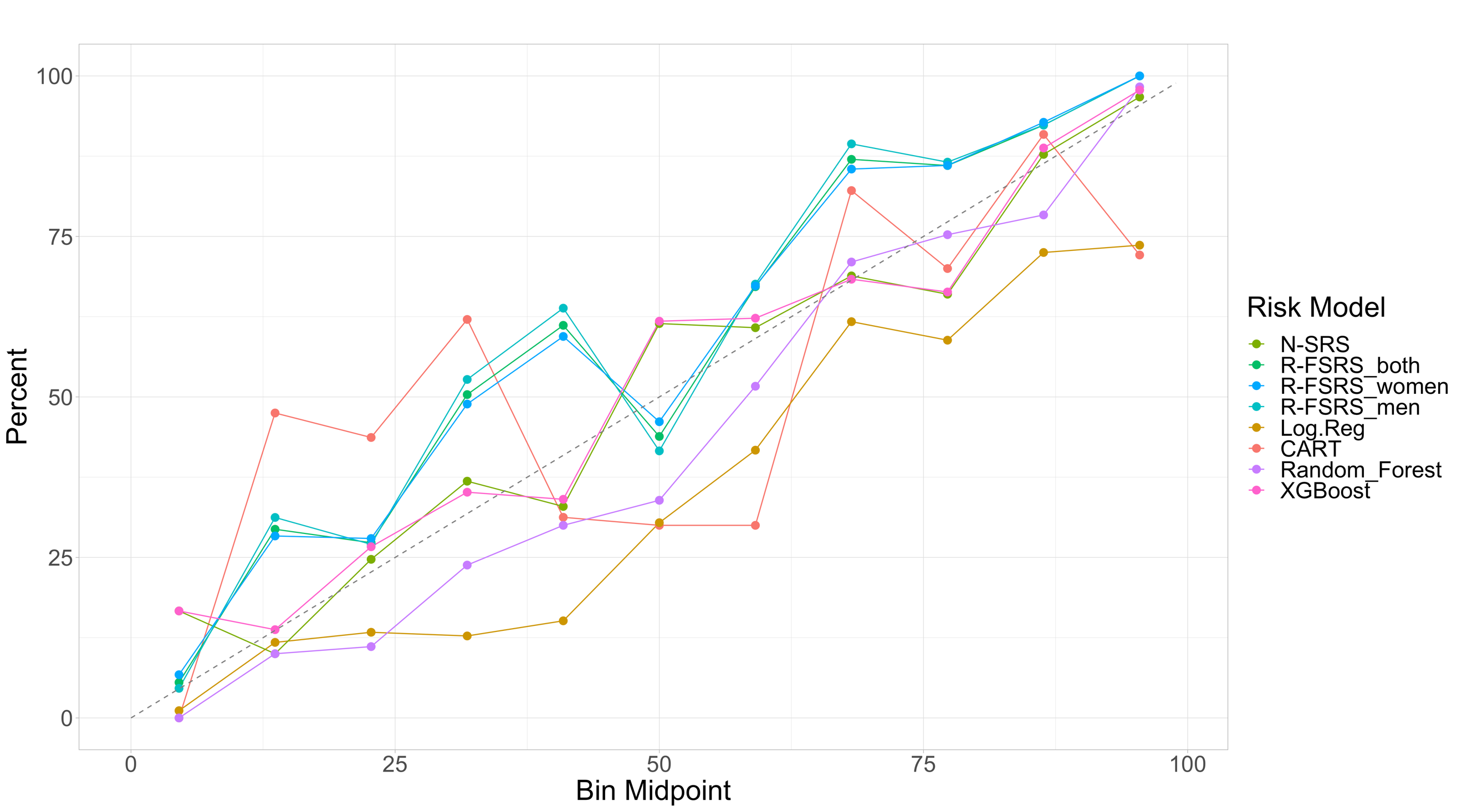


1. Calibration Curves for Framingham Dataset 1 (FD1).


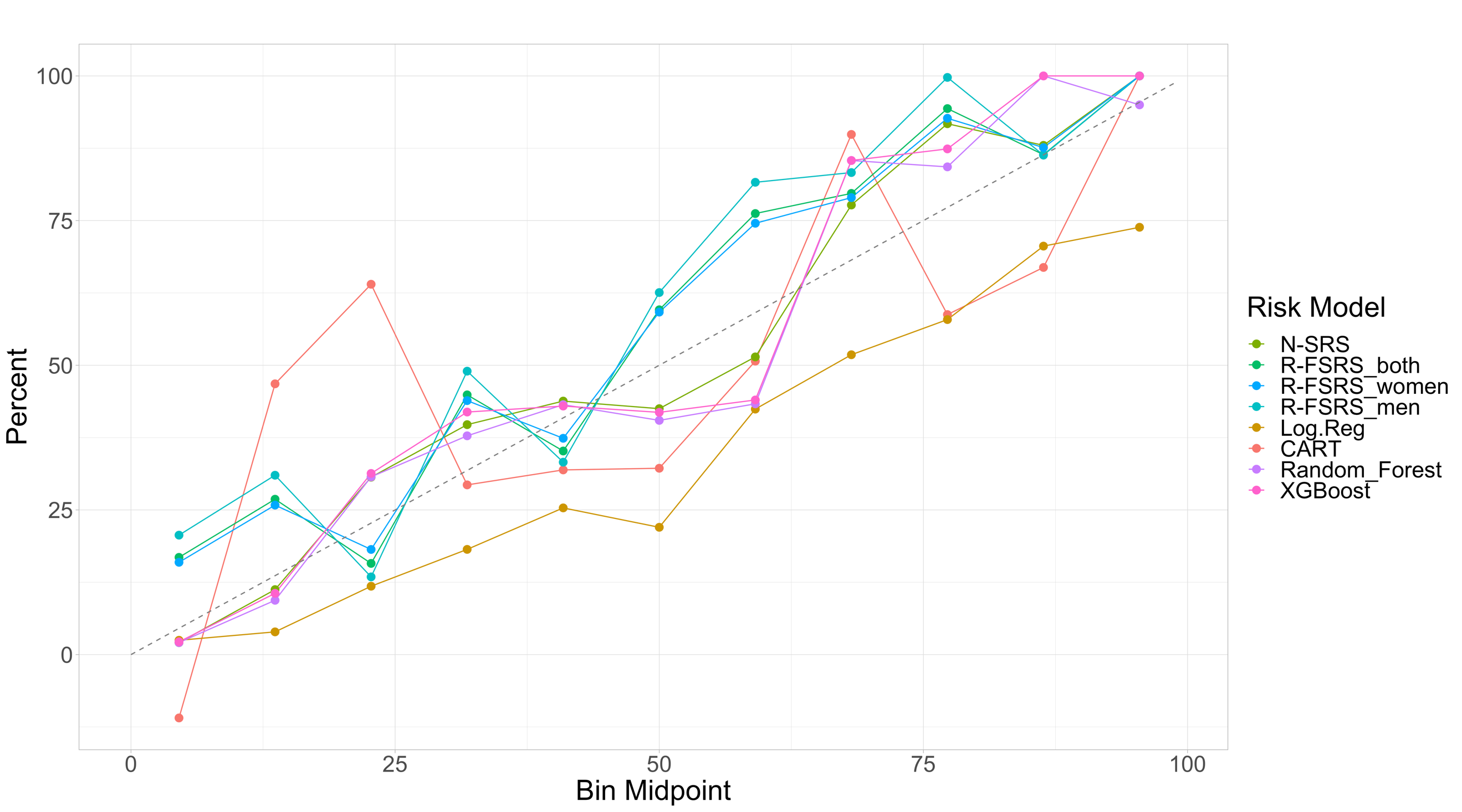


1. Calibration Curves for Framingham Dataset 2 (FD2).


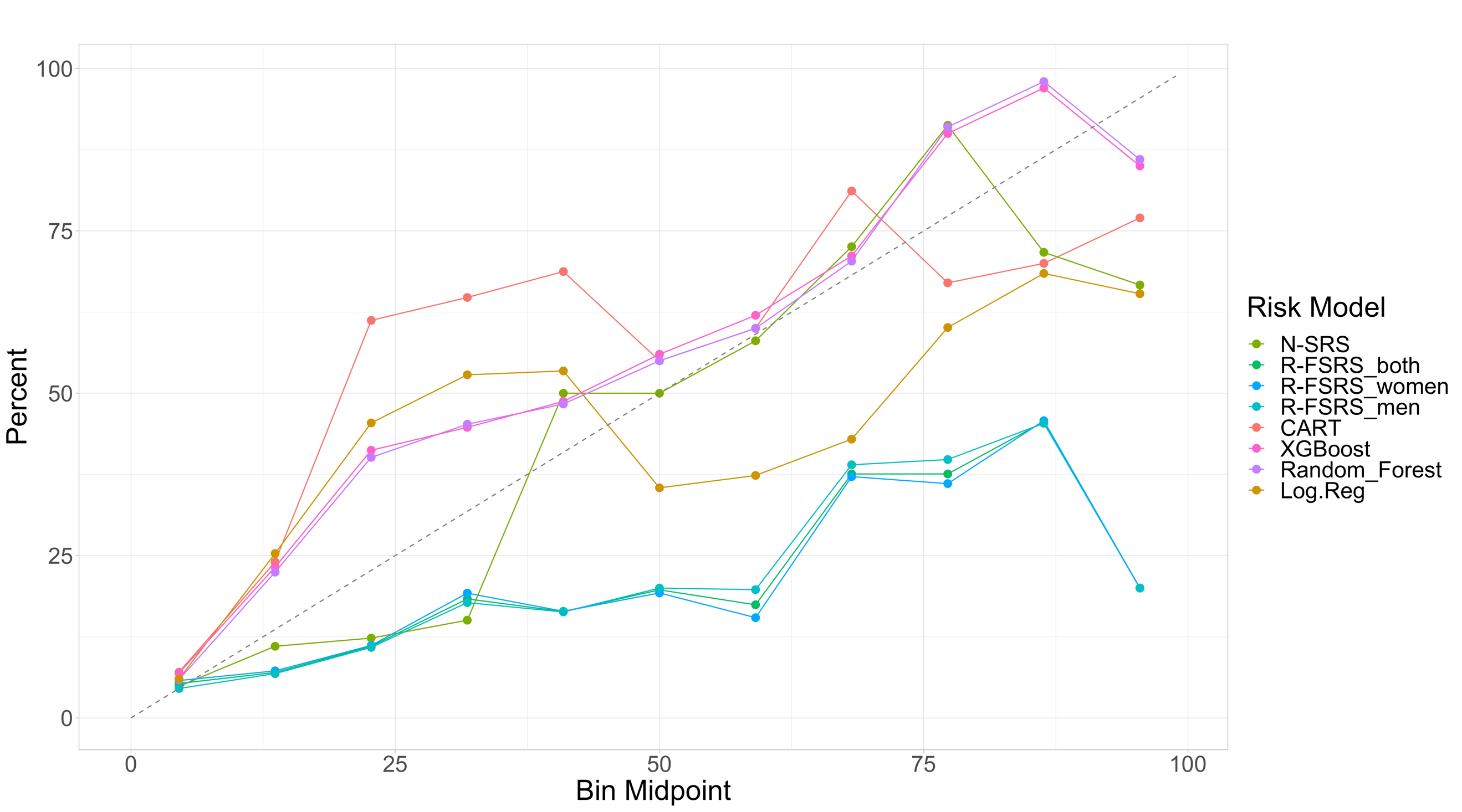


1. Calibration Curves for the BMC Validation Cohort.
