# Supplementary material for: Machine learning provides evidence that stroke risk is not linear: The non-linear Framingham stroke risk score
Source: PLoS One. 2020 May 21;15(5):e0232414. doi: 10.1371/journal.pone.0232414 (PMC7241753; doi:10.1371/journal.pone.0232414)
Supplement: S1 Table — (DOCX) [file pone.0232414.s003.docx]

**S1 Table: Prevalence of all Risk Factors at baseline examination for each cohort.**

| **Variable** | **FD 1 (train)** | **FD 1 (test)** | **FD 2** | **BMC All** | **BMC Black** | **BMC Caucasian** | **BMC Hispanic** |
| --- | --- | --- | --- | --- | --- | --- | --- |
| **Subject per sample, n** | 14195 | 4598 | 2989 | 17527 | 2862 | 9029 | 5636 |
| **Mean baseline Age** | 54.41 | 54.16 | 65.25 | 55.54 | 55.15 | 56.68 | 53.90 |
| **% Women** | 53.98% | 51.48% | 54.27% | 54.40% | 58.63% | 50.20% | 58.98% |
| **Currently smoking, %** | 25.62% | 26.49% | 13.62% | 34.71% | 38.04% | 37.86% | 27.95% |
| **Prevalent cardiovascular disease, %** | 9.00% | 9.33% | 17.55% | 20.85% | 27.88% | 19.77% | 19.01% |
| **Prevalent Atrial Fibrillation, %** | 1.39% | 1.54% | 2.97% | 2.13% | 1.23% | 2.29% | 2.34% |
| **History of Transient Ischemic Attacks, %** | 0.70% | 1.26% | 1.10% | 0.33% | 0.08% | 0.29% | 0.51% |
| **History of Myocardial Infarctions, %** | 2.86% | 3.04% | 4.72% | 0.45% | 0.47% | 0.41% | 0.52% |
| **Diabetes mellitus, %** | 5.78% | 7.66% | 9.47% | 6.91% | 7.89% | 6.54% | 7.02% |
| **Normal Blood Pressure status, %** | 29.64% | 29.64% | 22.82% | 14.60% | 11.02% | 15.37% | 15.20% |
| **Elevated Blood Pressure status, %** | 11.95% | 11.44% | 14.29% | 21.34% | 11.04% | 25.96% | 19.18% |
| **Hypertensive Blood Pressure status 1, %** | 41.90% | 42.39% | 41.45% | 43.37% | 45.56% | 40.28% | 47.20% |
| **Hypertensive Blood Pressure status 2, %** | 16.50% | 16.53% | 21.45% | 20.69% | 32.37% | 18.40% | 18.41% |
| **Antihypertensive medication, %** | 19.93% | 20.64% | 35.46% | 20.95% | 31.92% | 16.87% | 21.91% |
| **Statins, %** | 6.33% | 6.72% | 15.32% | 6.07% | 5.34% | 5.89% | 6.73% |
| **Nitrates, %** | 2.54% | 2.74% | 2.64% | 2.59% | 2.02% | 2.87% | 2.43% |
| **Diuretics, %** | 10.60% | 10.16% | 16.90% | 10.92% | 10.54% | 11.00% | 10.98% |
| **History of CABG, %** | 1.37% | 1.61% | 2.34% | 3.14% | 2.73% | 3.33% | 3.03% |
| **History of PCI, %** | 0.77% | 1.37% | 1.84% | 2.86% | 2.41% | 3.08% | 2.74% |
| **X-ray Enlargement, %** | 0.86% | 0.46% | 0.84% | 0.64% | 0.67% | 0.63% | 0.65% |
| **Left Ventricular Hypertrophy, %** | 2.33% | 2.11% | 1.20% | 5.41% | 5.14% | 5.54% | 5.34% |
| **Presence of T-Wave abnormality, %** | 12.36% | 12.07% | 14.55% | 12.75% | 12.54% | 12.87% | 12.65% |
| **Intraventricular Block, %** | 10.07% | 8.66% | 11.07% | 9.24% | 9.03% | 9.45% | 9.01% |
| **Atrioventricular Block, %** | 4.28% | 4.33% | 0.90% | 1.75% | 1.34% | 1.88% | 1.75% |
| **ST-Segment abnormality, %** | 9.70% | 9.94% | 14.22% | 17.51% | 15.67% | 18.39% | 17.03% |
| **U-Wave abnormality, %** | 6.70% | 6.57% | 6.62% | 6.54% | 6.57% | 6.52% | 6.55% |
| **Premature beats, %** | 4.48% | 4.74% | 5.89% | 6.57% | 5.99% | 6.85% | 6.42% |
| **Systolic Blood Pressure, mmHg** | 127.26 | 127.23 | 132.35 | 129.06 | 133.07 | 128.34 | 128.17 |
| **HDL mg/dl** | 51.19 | 50.23 | 51.91 | 51.69 | 53.40 | 52.72 | 49.18 |
| **BMI** | 27.17 | 27.39 | 27.81 | 30.17 | 31.46 | 29.47 | 30.64 |
| **Hematocrit** | 42.65 | 42.95 | 42.60 | 39.97 | 39.01 | 40.35 | 39.85 |
| **Fasting plasma glucose level mg/dl** | 99.82 | 101.59 | 102.36 | 117.10 | 121.52 | 108.73 | 128.26 |
