# Supplementary material for: Machine learning provides evidence that stroke risk is not linear: The non-linear Framingham stroke risk score
Source: PLoS One. 2020 May 21;15(5):e0232414. doi: 10.1371/journal.pone.0232414 (PMC7241753; doi:10.1371/journal.pone.0232414)
Supplement: S2 Table — The imputation accuracy was measured when the missingness percentage is fixed at 40%. Artificial values were introduced under the Missing Completely at Random pattern to measure the Mean Absolute Error (MAE) and the Root Mean Squared Error (RMSE). Predictive accuracy was assessed for an OCT model trained on the imputed data on the 10-year risk of stroke task. All metrics reflect the average value across five bootstrapped splits of DF1 in training and testing set. (DOCX) [file pone.0232414.s004.docx]

**S2 Table: Imputation algorithm comparison for the Framingham 1 dataset.** The imputation accuracy was measured when the missingness percentage is fixed at 40%. Artificial values were introduced under the Missing Completely at Random pattern to measure the Mean Absolute Error (MAE) and the Root Mean Squared Error (RMSE). Predictive accuracy was assessed for an OCT model trained on the imputed data on the 10-year risk of stroke task. All metrics reflect the average value across five bootstrapped splits of DF1 in training and testing set.

| **Metric** | **Mean** | **K-Nearest**  **Neighbors** | **OptImpute** | **MedImpute** | **MICE** |
| --- | --- | --- | --- | --- | --- |
| **MAE** | 1.9651 | 1.8342 | 1.6395 | 1.4441 | 1.5091 |
| **RMSE** | 6.7517 | 6.6231 | 6.0321 | 5.8342 | 5.9802 |
| **AUC** | 80.21% | 82.31% | 84.90% | 87.43% | 85.21% |
