# Supplementary material for: Machine learning provides evidence that stroke risk is not linear: The non-linear Framingham stroke risk score
Source: PLoS One. 2020 May 21;15(5):e0232414. doi: 10.1371/journal.pone.0232414 (PMC7241753; doi:10.1371/journal.pone.0232414)
Supplement: S3 Table — (DOCX) [file pone.0232414.s005.docx]

**S3 Table: Stroke Risk Factors identified in the N-SRS algorithm**

| **Demographic Factors** | Age |
| --- | --- |
|  | Gender |
| **Categorical Risk Factors** | Current cigarette smoking |
|  | Presence of Cardiovascular disease |
|  | History of Transient Ischemic Attacks |
|  | History of Myocardial Infarctions |
|  | Blood Pressure Category |
| **Medication and Treatment related Factors** | Antihypertensive medication |
| **ECG results** | Presence of T-Wave abnormality |
| **Continuous Risk Factors** | SBP |
|  | HDL |
|  | BMI |
|  | Hematocrit |
|  | Fasting plasma glucose level |
